# Towards community-based integrated care: trends and issues in Japan's long-term care policy

**DOI:** 10.5334/ijic.1066

**Published:** 2014-02-26

**Authors:** Mie Morikawa

**Affiliations:** Department of Health and Welfare Services, National Institute of Public Health, Wako-shi, Japan

**Keywords:** Integrated care, community-based, long-term care, care policy, Japan

## Abstract

**Introduction:**

In 2000, Japan implemented a mandatory long-term care insurance system. With the rapid growth of the system, problems became apparent. Several critical alterations were made to long-term care insurance system, particularly with respect to integrated care.

**Methods:**

This paper elucidates the policy trends that led to the reforms of the long-term care insurance system, which included new concepts of ‘integrated care’ and ‘community-based care’, an agenda of cost containment and service streamlining, and coordination with medical care.

**Results:**

Community-based integrated care, as envisaged in the long-term care policy, includes not only the integration of medical care into service provision but also the inclusion of the informal mutual aid, oversight of for-profit providers by an administration that ensures users are not exploited and coordination between systems that cover different geographical areas.

**Conclusions:**

Japan's experience in community-based care integration suggests that this project requires multi-faceted care integration in local communities. In the future, it will be necessary to conduct empirical assessments of the effectiveness of these measures.

## Introduction

The creation of a feasible scheme that guarantees long-term care is a challenge faced by the social welfare systems of all industrialized nations. In Japan, a mandatory long-term care insurance system was implemented in 2000, under the slogan ‘from care by family to care by society’. The intent of the long-term care insurance system was to prevent unnecessary hospitalization of seniors who had difficulties coping with everyday life by making institutional and home-based care available to every Japanese person aged 65 years and older, based on their physical and mental status [1–3].

In recent reforms to the system introduced in the Long-Term Care Insurance Act, national and local governments were given the responsibility of establishing community-based integrated care. 2012 was hailed as ‘year zero of community-based integrated care’ [4, p. 73]. Local governments are required not only to promote integrated care in their long-term care agenda but also to ensure that integrated care systems have been adopted by communities. As we will see in Section ‘The expansion of long-term care insurance system and the reform of 2005/6’, the concept of ‘community-based integrated care’ became the basis of long-term care policy soon after the inception of long-term care insurance system, which was heralded as offering ‘comprehensive care integrating the various resources of the community through coordination between formal health, welfare and medical care specialists, and further, including informal or mutual activities by the residents such as volunteers’. Integration of care was defined as follows: various forms of support are provided in a continuous and comprehensive manner in accordance with the situation of each elderly person, and changes in that situation, with the long-term care insurance services as the core.

The primary characteristic of the Japanese long-term care policy trend is evident in the name - community-based integrated care - which itself is a fusion of the two distinct concepts: integrated care and community-based care. Another prominent characteristic is that long-term care insurance, rather than the medical care system, is the driving force of the initiatives. Since its inception, long-term care insurance system has been constantly harassed by the need to respond to increases in costs brought about by a rapidly ageing population [5, 6]. When long-term care insurance system was originally established, it was separate from the medical insurance system; the recent integration of long-term care and medical care in service provision has important policy implications.

This paper will summarize the process by which the concept of integrated care was adopted by long-term care policy and the measures designed to promote that care, particularly the reforms of the long-term care insurance system in 2005/6 and 2011. Consideration will be given to the concepts of integrated care and community-based care, the agenda of cost containment and service streamlining, and coordination with medical care.

## The basic framework of long-term care insurance system

Municipalities are long-term care insurance insurers. This role came about naturally because the planners of the long-term care insurance programme conceived of long-term care as an extension of social welfare services, rather than an extension of health care services. Municipal governments had already accepted the responsibility for developing various types of social welfare services, as well as ‘community plans’ for the elderly. They were also the insurers for the national health insurance plan for self-employed and unemployed persons. It was therefore thought that municipal governments had sufficient experience to act as the insurers of long-term care insurance system [3, 7]. As there are several social insurance schemes offering medical services and the insurers are not limited to municipalities, it has been difficult to integrate long-term care and medical care in one insurance system at a municipality level. Also, the policies of the medical care insurance system made it difficult to integrate long-term care and medical care at the municipal level. The medical insurance system for the seniors aged 75 and over was enacted in April 2008 and is implemented by associations consisting of municipalities organized at prefectural level.

Once enrolled in the medical insurance plan, seniors can freely access medical services in either clinics or hospitals when these services are prescribed by doctors. Seniors’ access to long-term care services, however, must be approved by the municipalities. Certification of eligibility and determination of benefits in long-term care insurance system are based on a nationally standardized assessment process. Originally, six eligibility levels were established, but another was added in 2006. Each eligibility level entitles the applicants to a defined monetary allowance for services.

A contract-based system allows the beneficiary to choose among both services and providers of services. Formerly, citizens did not have a right to choose the type, amount, or provider of services. Users pay 10% of the costs and the remaining 90% is covered by long-term care insurance fees paid to the providers. For-profit organizations are allowed to participate as home-based service providers as long as they are approved by the local government. To be approved, they must meet the minimum standards for staff credentials and availability.

Care management services were institutionalized. The newly created occupation of ‘care manager’ required certification by prefectures. Care managers draw up care plans, coordinate the services for applicants according to their level of need, monitor their care and adjust care plans when necessary. Long-term care insurance covers the care management fee.

**Figure 1.**
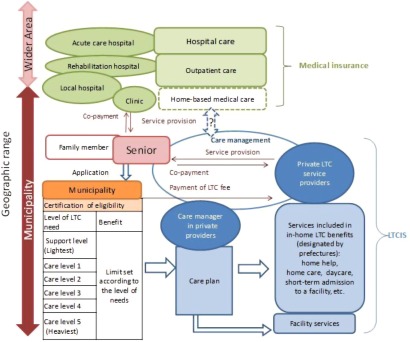
Structure of long-term care under the initial implementation of long-term care insurance system. Source: Originated by Author.

The structure of long-term care at the time of the initial implementation of long-term care insurance system is shown in Figure 1.

## The expansion of long-term care insurance system and the reform of 2005/6

### The problem of care management

Since the establishment of long-term care insurance system, the number of service users and the associated costs have increased rapidly. In 2000, when long-term care insurance system was launched, 21.7 million Japanese citizens were aged 65 or older, 2.2 million qualified for services, 1.5 million received services and 3.6 trillion Japanese Yen was spent on public care. In 2009, 28.4 million Japanese citizens were 65 or older, 4.7 million qualified for long-term care, 3.8 million received services and 7.7 trillion Japanese Yen (more than double the 2000 amount) was spent on public care. The number of seniors qualifying for long-term care and the number of users of the services have grown at a faster rate than the number of citizens over 65 [8].

As a result, there have been concerns about cost containment, which began not long after the introduction of the system. While low-cost home-based care was widespread, it did not supplant institutional care. The access to institutional care was quite limited: there were ‘insufficient facilities available’ for people who require long-term care. Institutional care was, however, inefficiently costly [9]. National policy-makers argue that simple quantitative expansion of the existing long-term care facilities will not ensure an efficient care system for Japan's ageing population. In order to contain costs, the national policy has been to improve and expand senior housing, and to increase the use of home- and community-based services, rather than invest in high-cost facilities.

In delivering home- and community-based services to senior citizens, care managers play an extremely important role. There has been, however, serious concern expressed over the care management they provide. While this problem has arisen as a result of a lack of skill and experience on the part of care managers [1], it has drawn attention to the structural weaknesses of long-term care insurance system itself.

Part of the problem is that long-term care insurance system has no clear division between care management and service provision, which leaves it open to abuse by care managers employed by profit-seeking private organizations. Unscrupulous care managers solicit users to employ services that are unnecessary but profitable. While there is no clear evidence that this kind of abuse has occurred on a widespread basis, there has been rising concern over administrative management [10]. A huge quasi-market for social care has arisen as a result of the admission of private for-profit providers in long-term care insurance system. Overseeing individual providers who lean too heavily towards profit maximization has become an important administrative task.

Furthermore, the long-term care system is not tailored to provide a comprehensive coordination of care resources. The duties of the care manager are not confined to creating a care plan under the auspices of long-term care insurance system. They should include developing and coordinating a wide range of care resources from health care to welfare, as well as various forms of informal support. Care managers, however, tend to confine their duties only to services covered by long-term care insurance. Before long-term care insurance system was launched, care resource management had been administered by local governments. With the inception of the long-term care insurance system, local governments shifted the responsibility to individual care managers, who have a narrower focus [11]. In response to these issues, the concept of ‘community-based integrated care’ was proposed during system reviews, which began three years after the inception of long-term care insurance system.

### Birth of ‘community-based integrated care’

In 2003, a research team set up by the Ministry of Health, Labour and Welfare produced a report entitled ‘Long-Term Care for the Elderly in 2015’. It identified mid-term and long-term goals for long-term care insurance system and elder care, and was to have a strong influence on the later direction of long-term care policy.

The report insisted a new long-term care service system was required, which would maintain the continuity of daily life for seniors requiring long-term care and allow them to remain in their neighborhoods. This would necessitate the establishment of a community-based integrated care system, which would provide multifunctional and composite services in homes and institutions. ‘Community-based integrated care’ was defined as ‘comprehensive care integrating the various resources of the community through coordination between formal health, welfare and medical care specialists, and further, including informal or mutual activities by the residents such as volunteers’, and the ‘community-based integrated care system’ was defined as ‘a mechanism by which various forms of support are provided in a continuous and comprehensive manner in accordance with the situation of each elderly person and changes in that situation, with the long-term care insurance services as the core [12]’. The report pointed out that, to ensure the functioning of the system, a central agency must be established to coordinate collaboration between the resources available.

The report argued that in order to maintain the continuity of a senior's daily life, community care resources management needed to be restructured. On the basis of the report, the Long-Term Care Insurance Committee of the Social Security Council published ‘Opinion on Revision of the Long-Term Care Insurance System’ in 2004, which proposed the establishment of a ‘community general support center’, under the responsibility of local municipalities (the insurers of long-term care insurance system), to provide comprehensive care management in the community [13].

### The 2005 reform of the long-term care insurance system

The 2005 reform of the long-term care insurance system introduced measures to contain costs, prevent abuses of services and strengthen coordinative efforts.

To contain costs, additional fees were imposed on users of facilities (such as assuming responsibility for hotel costs), and the benefits for those requiring little long-term care were reclassified as ‘prevention benefits’. Prevention benefits, which had lower monetary limits than other benefits, were applied to programs aimed at preventing further deterioration of health.

In order to prevent service abuses and strengthen coordinative efforts, community general support centres were set up by municipalities. Private non-profit organizations were often commissioned to administer the centres. Staff included three types of occupational specialists, certified social workers, public health nurses or registered nurses and care managers.

The care management for those eligible for prevention benefits was entrusted to the community general support centres rather than private care managers, to prevent exploitation on the part of the latter. To encourage coordination among various services providers, the centres were mandated to implement the following initiatives: the promotion of preventive self-care for seniors, comprehensive long-term care consultation, abuse prevention and rights advocacy, and support for continuous care management (e.g., guidance for care managers with difficult cases). Centre staff are also expected to encourage the use of community resources and networking among resource providers [14, 15]. By 2006, there were 3436 community centres in Japan representing 1483 long-term care insurers (87.8%). By 2008, all insurers had established centres, and by 2011, the number of centres reached 3976 [16].

**Figure 2.**
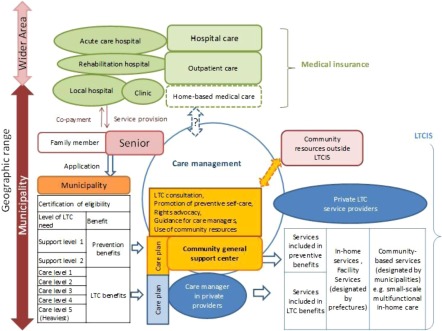
Structure of long-term care under the reform of 2005/6. Source: Originated by Author.

The structure of long-term care envisaged under the reform of 2005/6 is shown in Figure 2.

## Towards a ‘community-based integrated care system’ and the reform of 2011/12

### ‘Community-based integrated care system’ as a basic principle

Ten years after the inception of the long-term care insurance system, a lively policy debate took place regarding issues arising from the promotion of community-based integrated care. The reports published by the Community-Based Integrated Care Research Committee in 2009 and 2010 on the outcomes of the projects commissioned by the Ministry of Health, Labour and Welfare provided the main impetus for the debate [17, 18].

The reports laid out the goals for the system with a projected completion date of 2025. These goals included coordination between long-term care and health care workers, housing authorities and community-based informal service providers. Proposals were offered to achieve the target. In the fiscal year 2008 report, the ‘community-based integrated care system’ was newly defined as ‘the system of the community that, having provided housing in accordance with needs as the basic function, can appropriately offer each elderly person not only health care and long-term care but also various forms of support services, in order to ensure safety, security and health in their everyday life [17, p. 6]’. Users should be able to expect quick responses to any critical situation (within thirty minutes) and the availability of community services 24 hours a day, 365 days a year. On the basis of this target, the long-term care reforms of 2011/12 were introduced.

### The agenda of the long-term care reforms of 2011/12

Given a limited budget, the agenda of the long-term care reforms was to provide an effective and efficient system, while keeping costs down [19].One of the main items of the agenda was the improvement of home-based care, including medical care. With the expected increase in households composed solely of seniors, the demands on long-term care were bound to grow significantly in the near future. For cases where users needed both long-term care and medical care, a system was proposed whereby the users could live at home for as long as possible and the length of any hospital stay would be minimized. Various suggestions were considered, including the instigation of a 24-hour system of home-based care with coordinated medical care that would be capable of dealing with a range of medical situations from chronic ailments to advanced acute illnesses.

Also on the agenda was the systemization of diverse social resources for care. In order to make it possible for elderly persons to continue to live in the community, it was necessary not only to enhance coordination between the services covered by medical insurance and long-term care insurance but also to put in place various forms of daily support. Mobilizing social resources would provide comprehensive support, including medical treatments, long-term care, health services, daily life support and housing. To contain costs, priority was given to those requiring intense long-term care. Those requiring light social care would be placed outside the scope of insurance beneficiaries and their services handled ‘outside’ long-term care insurance. Instead, they would rely on community mutual aid resources.

Integrated care was, therefore, something that required coordination not only of different formal services of publicly financed systems but also of the various resources in the community. Concrete measures to promote integrated care included the establishment of community general support centres and the assumption of management functions on the part of the local government. In response to this policy agenda, long-term care insurance system was reformed in 2011, leading to the new services and the local administration strategy implemented in 2012 [20].

### New long-term care services for promoting integrated care

Two main initiatives were introduced in 2012. The first was regular home visits and as-needed long-term care and nursing visits, which required close collaboration between long-term care and nursing services. There are regular visiting patrols both day and night, and quick response visits on an as-needed basis. The second initiative was the creation of a composite service, which added health care to the ‘small-scale multifunctional in-home care’, which was established in 2006. Both initiatives are ‘community-based’ services: with the funding of long-term care insurance, the local administration hires a service provider for a fixed monthly fee. These newly introduced services play an important role in supporting the daily home lives of people with intense long-term care and health care needs.

The long-term care and nursing staff responsible for home visits work from one location in order to provide stable, yet flexible, service. These home visits were proposed when it was evident that, as the user's need for long-term care grew, nurses and health care workers would be required, often only for a short time, to maintain daily life at home. Under the earlier regime of long-term care home visits, only a small number of visits were made per day. This made it difficult to provide medical care and nursing on an as-needed basis [20].

The second initiative arose from the fact that the ‘small-scale multifunctional in-home care’ service created in 2006 had encountered certain problems. It was assumed that the users would require relatively intense long-term care, roughly equivalent to those living in facilities. However, many people failed to register as users, so the average long-term care requirement was considerably lighter than originally anticipated [20]. Better health care and nursing functions were necessary in order to make the service easier for people with intense long-term care requirements.

### A new strategy for the management of long-term care insurance system and integration of care resources at the local level

Ministry of Health, Labour and Welfare, in reviewing long-term care insurance system for the sixth municipal plan, has taken a keen interest in the role of local administrations in the management and integration of long-term care insurance and other social resources. Regarding long-term care insurance management by municipal administrations, Ministry of Health, Labour and Welfare has proposed conducting surveys of the needs of elderly households and tailoring services to particular communities rather than the entire municipality. Since almost all long-term care insurance system services are carried out by private providers, it is necessary to evaluate their performance and determine if there are any discrepancies between the actual services offered and those outlined by the municipal administration. The Japanese government anticipates that a repetition of the Plan-Do-See cycle at three-year intervals will take root in local administrations [4].

After the 2005 system reform, there were high hopes for the integration of long-term care insurance services and other necessary care resources at the local level. It was assumed that the community general support centre would act as an effective coordinator. Unfortunately, the centres were not very successful in coordinating services [21, 22]. The revised Long-term Care Insurance Act of 2011 added the following responsibility to the roster of the community general support centres: the local municipalities responsible for establishing the centres ‘must strive to coordinate with long-term care service providers, commissioned welfare volunteers, volunteers involved with activities supporting the daily lives of elderly persons, and other related persons’ (Paragraph 46, Article 115 of the revised Long-term Care Insurance Act). This clearly included coordinating activities in the official duties of the centres. The ‘Outline of the Establishment and Management of Community General Support Centers’, amended to reflect the revised long-term care insurance Act, expressly stipulates that centres should create networks for long-term care-related organizations and persons, and encourages the establishment of a Community Care Council [23].

Community Care Councils support community networks by arranging for discussions of individual cases, which outline each user's care management programme and identify any problems. The Council includes staff from the municipality, the centre and long-term care-related organizations. Separate councils can be set up for various regions within the municipality or for a collection of municipalities if that would best serve the community. The outline stresses the importance of close coordination of medical treatment and long-term care. In addition, it recommends that municipalities create a mechanism for sharing user data among support providers, while keeping in mind the need to protect personal information [23].

### New direction for the integration of the medical care and long-term care systems

At the same time as efforts for the full-fledged construction of the comprehensive community care system were under way, health care policy guidelines were developed to review hospital care and home-based medical care and to promote coordination between medical care and long-term care.

In the late 2000s, the regional five-year health plans drawn up by the prefectures aimed to integrate the various providers - primary care practitioners, acute care hospitals, rehabilitation hospitals, long-term care facilities and home-based care [24]. The five-year plan of fiscal year 2013 specified that achievement targets should be incorporated in home-based medical care and that these targets should be interlinked with the long-term care insurance schemes [25]. Monetary incentives were offered to realize these policy aims. Early discharge from hospital for elderly patients and home-based medical treatments were encouraged by a new structure of fees under the medical insurance. The new fees supported the coordination of care managers in hospital discharge support and increased the compensation to doctors who advised care managers in home-based care.

In 2012, the budget to promote community initiatives for home-based medical care was increased to 20 times that of the previous year. Model projects coordinating various means of home-based medical care have been implemented in 105 locations nationwide. In the model projects, coordination is based not in the community general support centres, but at local hospitals, clinics or other medical institutions. The bases are staffed by specialists who are well-versed in the fields of medical care and long-term care. The staff facilitates smooth hospital discharge by instituting information-sharing between primary care practitioners and hospitals, collaborating with the community general support centres, and keeping an eye out for new resources that would be useful in further collaboration [26].

**Figure 3.**
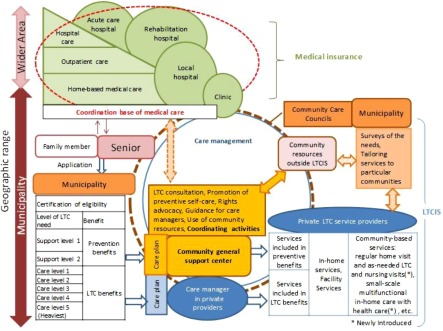
Structure of long-term care under the reform of 2011/12. Source: Originated by Author.

The Structure of long-term care envisaged under the reform of 20011/12 is shown in Figure 3.

## Discussion

Thus far we have outlined how, in recent years, community-based integrated care has become part of the long-term care policy agenda, as well as trends in the long-term care insurance system and health care system for integrated care. Given governmental concern with cost containment, the encouragement of community-based integrated care systems has been premised on the notions that, first, the care is provided from a range of resources, including mutual aid and self-help, and, second, that those who need intense long-term care will be given priority in the use of formal system resources. Based on these assumptions, new services with enhanced health care functions have been introduced to long-term care insurance system. Reforms aimed at the expansion of home-based medical care, combined with monetary incentives for medical and long-term care collaboration, have been initiated. The long-term care insurance insurers - the municipalities - have been required to show a positive commitment to the management of care resources within the community and to coordinate overlapping services.

To answer the questions set out at the beginning of this paper, we must look at the background of the movement towards community-based care. When long-term care insurance was introduced, the long-term care system became independent of the medical care system, offering primarily social care and welfare services. Later discussions of integrated care did not focus exclusively on collaborations with medical professionals; they also were intended to promote the coordination of all social care resources available to support continuity of the daily lives of seniors. In social services, informal care is much more common than it is in medical services. In order to contain the costs associated with medical attention, the integration of informal care became a significant element of the policy agenda in the promotion of community-based integrated care. With an ageing population, however, the greater part of long-term care insurance benefits is gradually shifting towards more intense care services. From now on, social care will be increasingly redefined as an important resource but something that is secured ‘outside’ long-term care insurance services. For that reason, the pressures placed upon the mutual aid facilities of communities will also continue to be on the rise. Unfortunately, we cannot say that effective methods for coping with these pressures have been established.

Long-term care insurance system adopted the ‘quasi-market mechanism’ as its service model. Under the quasi-market mechanism, profit-seeking providers have increased the risk of superfluous services. Municipalities were required to take measures to solve this risk such as replacing private providers with community general support centers in the role of care management and requiring insurers to develop long-term care insurance plans based on needs surveys. The Community Care Council has been introduced to share the community-based care mission with providers. Such efforts may counteract the threats associated with commercialization, though this remains to be seen.

Attempts to integrate community-based care and long-term care require coordination with the medical care system. Municipalities must now manage the Community Care Council, construct a providers’ network and include medical care providers within that system. The care integration within the medical system, however, includes coordination between clinics located in a municipality and hospitals providing care for a wider territory. Casework, therefore, often must cover a wider geographic range and is not confined to the municipality. In this respect, the attempt to include medical care into the integrated care network is at odds with the goal of creating small-area care system. In order to coordinate systems that cover different geographical areas, there is a plan to effect a collaboration between the community general support centre and a coordinating body in medical care field. The effectiveness of such a collaboration is still to be determined.

In an effort to realize community-based integrated care, the government is asking municipalities to construct the consultation and networking apparatus necessary for that purpose. Given the example of joint commissions of health and social care in other countries such as England [27], it would seem that there is still room for argument about how effective these methods of integrated care really are. Given the multifaceted issues outlined above, it is still critical to examine of the outcomes of care integration and the mechanisms used to effect it.

## Conclusion

Japan's long-term care policies for the elderly aim to create multifaceted care integration in local communities. They respect the value of continuity of life in the community for the elderly, as well as the economy of such measures. Japan's experience in community-based care integration suggests that this project requires the integration of social care and medical care, the inclusion of informal mutual aid, administrative restraints on profit-seeking and coordination between systems with different geographical spheres. In order to determine the effectiveness of these initiatives, empirical assessments are necessary.
